# Tinea capitis among schoolchildren in Ethiopia: A systematic review and meta analysis

**DOI:** 10.1371/journal.pone.0280948

**Published:** 2023-02-10

**Authors:** Molla Yigzaw Birhanu, Habtamu Temesgen, Daniel Bekele ketema, Melaku Desta, Temesgen Getaneh, Getamesay Molla Bekele, Balew Zeleke, Selamawit Shita Jemberie

**Affiliations:** 1 Department of Public Health, College of Health Sciences, Debre Markos University, Debre Markos, Ethiopia; 2 Department of Human Nutrition and Food Science, College of Health Sciences, Debre Markos University, Debre Markos, Ethiopia; 3 Department of Midwifery, College of Health Sciences, Debre Markos University, Debre Markos, Ethiopia; 4 Department of Gynecology and Obstetric, School of Medicine, Debre Markos university, Debre Markos, Ethiopia; 5 Department of Pediatrics and Child Health Nursing, School of Health Sciences, College of Medicine and Health Sciences, Bahir Dar University, Bahir Dar, Ethiopia; Gulu University, UGANDA

## Abstract

**Background:**

Tinea capitis accounts for25 to 30% of all fungal infections, but it is often ignored because it is not life threatening in nature. It is more common among schoolchildren particularly in developing countries. Due to the presence of significant variability among the previous studies, this study was conducted to provide a pooled prevalence and associated factors of tinea capitis in Ethiopian schoolchildren.

**Method:**

We conducted a systematic search in five major databases for articles similar to our topic. This review included school-based cross-sectional studies that were reported in English and conducted from 2006 through 2022. The data were extracted using Microsoft Excel and further analysis was done using Stata^TM^ Version 17.0 statistical software. Forest plots were used to assess the presence of heterogeneity with 95% confidence intervals. A random effects meta- analysis model was used to pool primary estimates. To declare the presence or absence of association, 95% confidence interval with odds ratio was used.

**Results:**

Fourteen studies with a total of 9465 schoolchildren were included. The pooled prevalence was 29.03% (95%CI: 15.37–42.71). There was observed heterogeneity, which could be explained by publication bias (P = 0.04). Family history of tinea capitis (OR: 9.18, 95%CI: 3.5–24.02), under the age of 10 years (OR: 1.65, 95%CI: 1.17–2.33) were factors increasing the development of tinea capitis among schoolchildren and schoolchildren who had hair wash at least once a week (OR: 0.31, 95%CI: 0.24–0.42) was significantly associated with reduced risk for tinea capitis.

**Conclusion:**

One of the most prevalent childhood health condition in Ethiopia is tinea capitis, which affects over one in every four schoolchildren. Schoolchildren who had family history of tinea capitis and under the age of 10 years were the identified risk factors but they had hair wash at least once a week was the protective factor of tinea capitis among schoolchildren. Clinical and public engagement activities are needed to overcome the burden of the disease.

## Introduction

Tinea capitis is a fungal infection of the hairs on the scalp, eyelash and brows [1]. It is caused mostly by Trichophyton and Microsporum that can infect keratin, keratinized tissue, and hair [2]. It affects people of all ages, but children aged from 3 to 14 are the most affected [3,4]. It is responsible for up to 92.5% of all dermatophytoses in children under the age of ten [5,6]. It can be diagnosed clinically and using laboratory investigation. Hence, to identify tinea capitis from its differential diagnosis, clinicians used different diagnosising approach of tinae capitis like clinical (history taking and physical examination) as well as laboratory investigation (wet mount and culture). It is clinically presents as single or multiple scaly alopecia patches and alopecia patches with black dots at follicular orifices representing broken hairs, a little erythematous papule around a hair shaft on the scalp, eyebrows, or eyelashes are the most common clinical findings [7]. It can be inflammatory or non-inflammatory, with the inflammatory type causing kerion (painful pus-filled nodules) and scarring alopecia [8]. Definitive diagnosis can be made by demonstrating the presence of fungal elements via potassium hydroxide (KOH) wet mount or isolating of pathogenic fungi on appropriate media. KOH wet mount has a high sensitivity but low specificity and may be the only available technique. Addition of fluorescent brighteners such as calcofluor white may improve the demonstration of fungal elements. Fungal culture is the gold standard and has a better specificity with the added advantage of allowing for antifungals susceptibility testing, to influence the drug of choice for treatment [9]. In recent times, genomic and proteomics techniques such as polymerase chain reaction (PCR) and matrix-assisted laser desorption and ionization time of flight (MALDI-TOF) play a significant role in identifying rare etiological agents [10,11]. The most and second common agent aisolated for tinea capitis were Microsporum cani and Trichophyton rubrumin in Korea [12], Trichophyton verrucosum and T. tonsurans in Ethiopian schoolchildren respectively [13].

Tinea capitis is treated using antifungal and the drug of choice is determined by the strain of tinea, the degree of inflammation, and the patient’s immunologic and nutritional status [12]. Nowadays, antifungal resistance is increasing, making patient management more difficult [14]. To prevent this resistance, antifungal susceptibility tests, particularly for difficult-to-treat infections, should be performed to determine the best drug of choice and dosage [15]. Antifungal sensitivity tests conducted previously revealed that amphotericin was the most effective antifungal agent, followed by terbinafine and itraconazole [16,17]. It is a highly contagious disease that can transmitted from another human or an animal through direct contact and most prevalent in rural areas. Poor personal hygiene, overcrowding, under the age of 10 years, male gender, and a low socioeconomic status are all factors that contribute to the spread of the infection [1,18].

As the previous studies revealed that the prevalence of tinea capitis among schoolchildren in Ethiopia is high with significant variation and prevention and treatment efforts of the disease has not improved [19–21]. As a result, the goal of this study was to determine the pooled prevalence and associated factors of tinea capitis among schoolchildren in Ethiopia. The findings of this study may lay the scientific groundwork for a better appreciation of the updated epidemiology of Tinea capitis in Ethiopia to guide the development of effective preventive and management approach.

**Research question**: what is the prevalence and associated factors of tinea capitis among Ethiopian primary schoolchildren?

**Condition:**Tinea capitis

**Context:** Ethiopia

**Population:** Primary schoolchildren in Ethiopia

## Methods

### Study area

**Fig 1:**
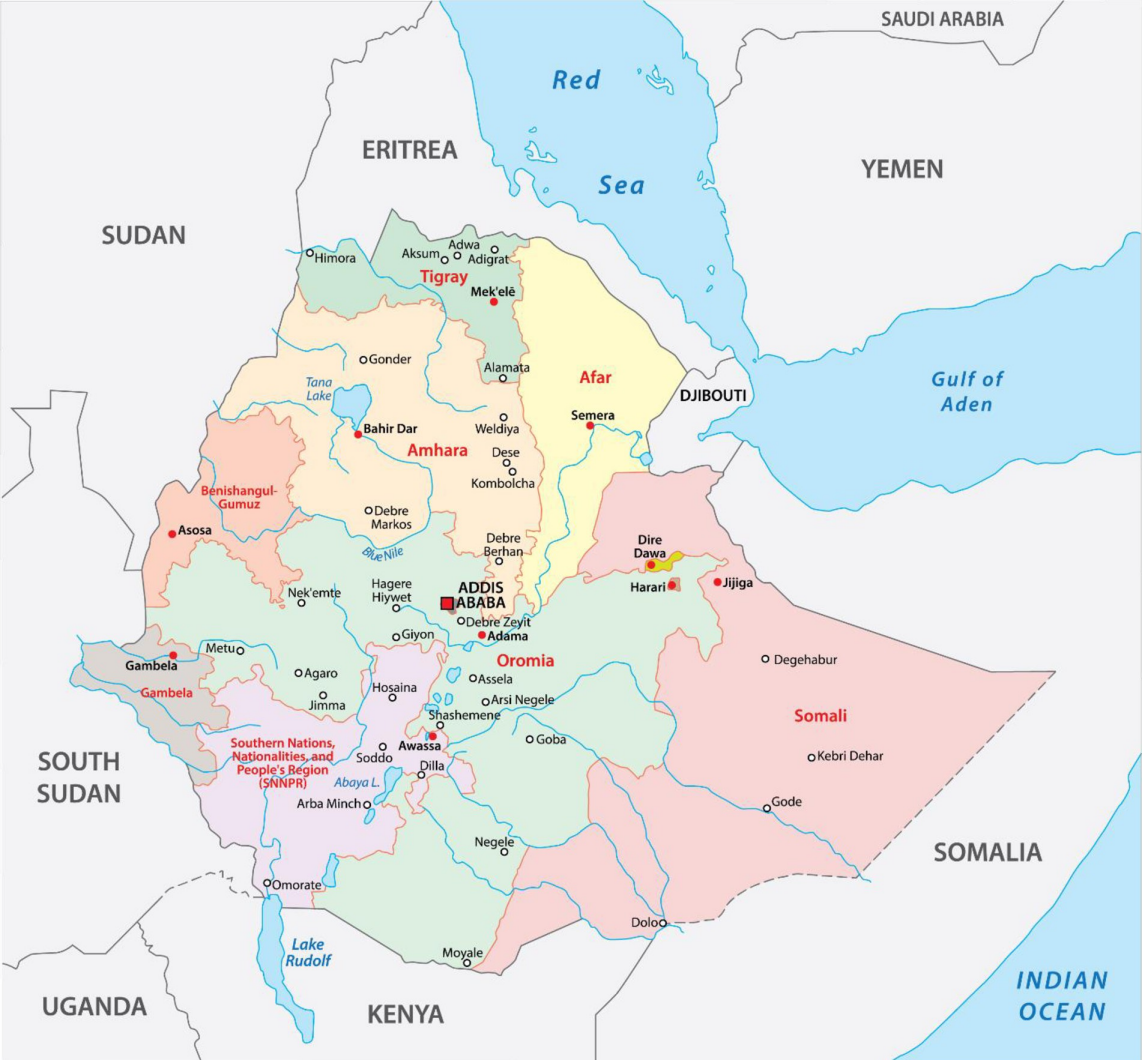
PRISMA flow diagram of the included studies for tinea capitis among schoolchildren in Ethiopia.

This study was carried out in Ethiopia which is organized as a Federal Democratic Republic with nine regional states (Afar, Amhara, Benishangul-Gumuz, Gambella, Harari, Oromia, Somali, Southern Nations Nationalities and People’s Region, and Tigray) and two city administrations (Addis Ababa and Dire Dawa). It covers an area of 1,100,000 km^2^, and its regional states are divided into zones, which are further subdivided into districts, which are further subdivided into kebeles, the lowest administrative divisions [22]. Ethiopia is the second most populous country in Africa with a population of about 112 million people (56,010, 000 females and 56, 069, 000 males in 2019) [23] (Fig 1).

### Data source and searches

The Preferred Reporting Items for Systematic Review and Meta Analysis (PRISMA) guideline was used to report this review [24]. (S1 File). We systematically searched five major databases (PubMed/MEDLINE, CINAHL, EMBASE, Google Scholar, and Science Direct) for previous studies similar to our topic. Additionally, important articles were found by searching the reference lists of eligible studies. The search was carried out independently by three authors (MYB SSJ, and HT). Endnote X9 was used to retrieve and manage studies found through a systematic search. The following terms were used in the above-mentioned databases searches: "tinea capitis" OR "prevalence" AND "schoolchildren" OR "associated factor" AND "Ethiopia." The searching strategy was began in 28 June 2022 up to 20 July 2022.

### Study selection criteria

**Inclusion criteria.** All studies conducted on tinea capitis among Ethiopian schoolchildren were included.

**Exclusion criteria.** Articles were excluded when they were not fully accessed after two email contacts with the correesponding authors.

**Design**: Systematic review and Meta analysis

**Publication status:** All published and unpublished articles

**Language**: English language

### Screening procedure

All titles/abstracts found in electronic databases were independently screened by five authors (MYB, GMB, MD, DBK, SSJ). Disagreements were settled through discussion. Four authors (MYB, SSJ, TG, GMB) independently screened the full text of screened and included articles for title and abstract. Disagreements were settled through discussion. In the event of a disagreement, the third author (HT) was consulted.

### Data extraction process and quality assessment

The data were extracted using the data extraction Cheklist prepared from a Microsoft Excel spreadsheet. To ensure consistency, three authors (MYB, SSJ, and DBK) extracted data independently using a predefined extraction checklist. After the source of the disagreements was identified, disagreements between or among authors were resolved through discussion. The prevalence of tinea capitis in schoolchildren, the study setting, region, year of publication, sample size, and the first author name were all the extracted data from the primary article during extraction.

The Newcastle-Ottawa Quality Assessment Scale (NOQAS) [25] was used to evaluate the quality of the included primary studies based on study representativeness, adequate sample size, acceptable non-response rate, use of validated measurement tool, comparability of the study, description of outcome assessment, and use of appropriate statistical tests as parameters. During the quality assesment of articles, the articles that scored seven out of ten were declared as high-quality article [26,27] (S2 File)

### Outcome variable and measures

In this systematic review and meta analysis, prevalence of tinea capitis was the main outcome of interest. It was calculated by pooling the prevalence of tinea capitis among Ethiopian schoolchildren from previous studies. The second outcome of interest was associated factors of tinea capitis and measured by computing binary meta regression using random effects meta regression model with 95% confidence level and p-value < 0.05.

### Data management and synthesis

The extracted data were exported to Stata^TM^ Version 17.0 software for further analysis. The metaprop stata command was used to compute the pooled estimate. Using a binominal distribution assumption, the standard errors were calculated from the reported estimates and population denominators.The presence of heterogeneity between studies was checked using Cochran’s-Q test and quantified using I-square statistics and significant heterogeneity was found. Thus, a random-effect model based on DerSimonian and Laird was used to estimate the effect size [28]. Accordingly, heterogeneity was classified as low, moderate, or high when the values of I-square were 25, 50, and 75%, respectively [19]. Additionally, the dispersion of individual results in the forest plot was also used to evaluate the presence of heterogeneity visually. Egger’s linear regression test at a p-value < 0.05 was used to assess the presence of publication bias [20]. A 95% confidence interval was used to calculate and report an overall synthesised measure of effect size.To identify the possible sources of heterogeneity, additional statistical analyses such as subgroup analyses, publication bias, and meta-regression were performed. We conducted a subgroup analysis using geographical regions, the study setting (urban Vs rural), sample size (above mean Vs below the mean), and study period(before 2015 Vs after 2015). Finally, the results were presented in tables and forest plots.

### Patient and public involvement statement

Patients were not directly involved in the study, only previously conducted studies were employed. Hence, new study participants were not recruited and participants were not involved in the dissemination of findings.

## Results

### Search results

**Fig 2:**
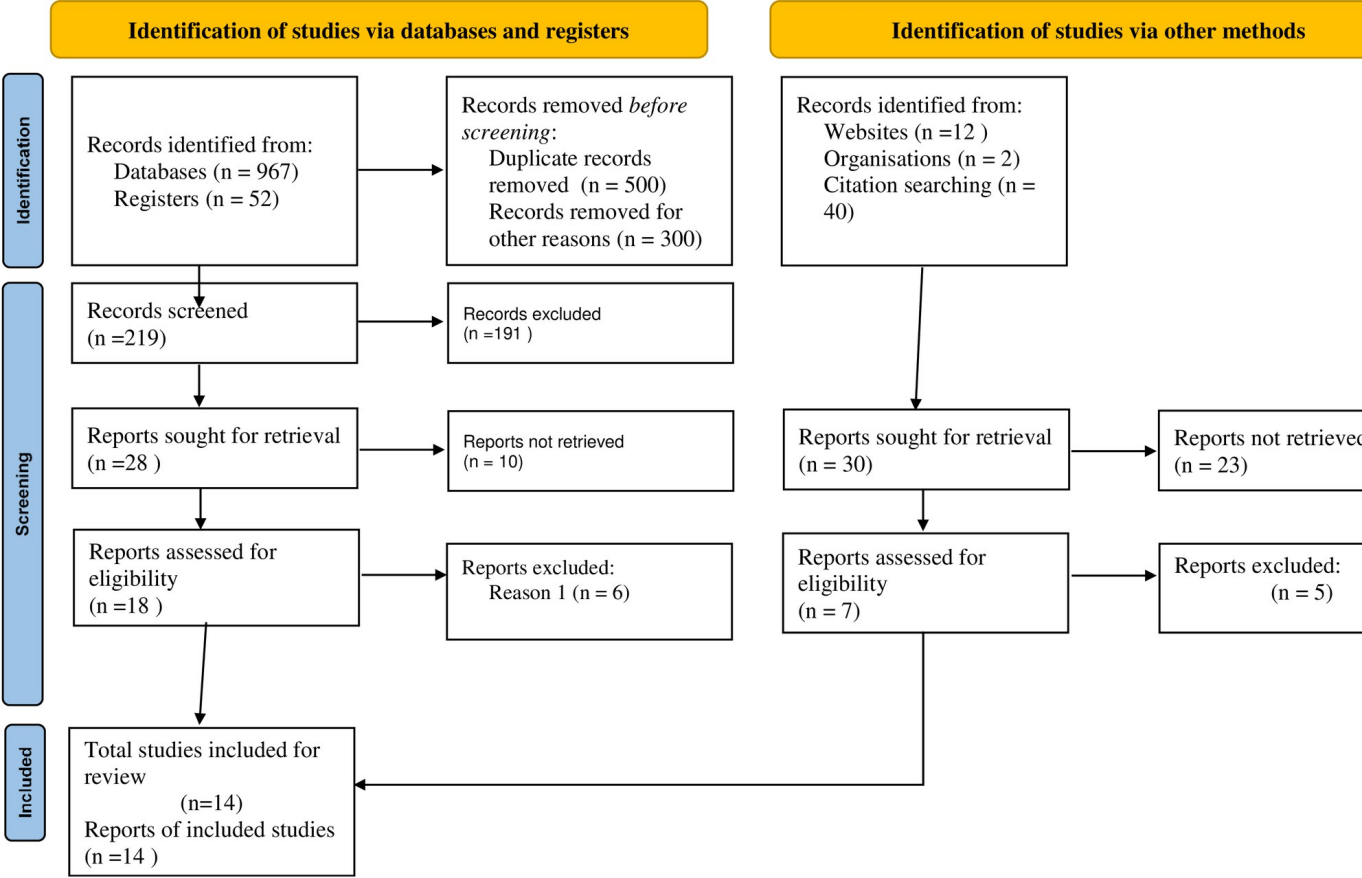
The subgroup analysis of tinea ccapitis over region in Ethiopia.

A total of 1073 studies were found through electronic and other searches (like organizational records and websites) on PubMed/ MEDLINE, CINAHL, EMBASE, Google Scholar, and Science Direct. About 824 articles were excluded due to duplication, 191 articles were excluded due to different in study setting/context [29–32], 33 articles were excluded due to different in interest of outcome [33–38], and 11 articles were excluded due to different in study population [39–41]. Finally, 14 cross-sectional studies were identified to be included in the current systematic review and meta-analysis (Fig 2).

### Characteristics of included studies

About 14 studies qualified for inclusion and analysis with a total of 9465 primary schoolchildren in Ethiopia.This meta-analysis and systematic review included three regions and one city administration. Among them were the Amhara region (n = 4) [20,42–44], the Oromia region (n = 4) [13,19,45,46], the SNNPR (n = 3) [21,47,48], and Addis Ababa (n = 3) [49–51]. About 5 studies out of the total included studies identified the causative agent of tinea capitis, *T*. *violaceum*, and the rest did not identify the species. Five of the included studies used culture to diagnosis and identify the species, while the others used clinical and KOH to diagnose and identify the causative agent. The study with the smallest and largest sample sizes, 288 and 1869, both conducted in the SNNPR’s Hawassa and Wolita zone, respectively, followed by a study with a sample size of 1122 in Merkato, Addis Ababa’s administrative city (Table 1).

**Table 1:** The characteristics of the studies included in tinea capitis among Ethiopian schoolchildren.

Sn	Authors	Publication year	Region	Study area	Sample size	Prevalence
1	**Adane Bitew *et al*** [49]	2021	Addiss Ababa	**Wollo Sefer**	364	82.7
2	Tizazu Getahun *etal* [42]	2022	Amhara	Finoteselam	370	18.4
3	R Perez-Tanoira *et al* [13]	2017	**Oromia**	**Gambo**	634	15.6
4	**Yohannes Lulu *et al*** [46]	2017	Oromia	**Illu aba bora**	828	9.2
5	**Hiwot Hailu Amare *etal*** [47]	2021	SNNP	**Gedeo**	864	74.6
6	Feleke Moges *et al* [43]	2010	Amhara	Gondar	870	8.2
7	Maria Leiva-Salinas *et al* [52]	2015	Oromia	Gambo	647	8.7
8	Desalegn Tsegaw Hibstu *et al* [48]	2017	SNNP	Hawassa	288	32.3
9	**Shambel Araya *et al*** [50]	2021	Addiss Ababa	Merkato	1122	24.1
10	**Y. Woldeamanuel *et al*** [51]	2006	Addiss Ababa	Alert	374	**69**
11	**Sora Asfaw Desisa *et al*** [44]	2019	Amhara	**Gondar**	405	**21.7**
12	**Alem Alemayehu *et al*** [19]	2016	Oromia	**Harari**	428	**18**
13	**Anteneh Mengist Dessie *et al*** [20]	2022	Amhara	Debre Berhan	402	**11.8**
14	Abraham Getachew Kelbore [21]	2019	SSNP	**Wolaita**	1869	**12.7**

### The pooled prevalence of tinea capitis

**Fig 3:**
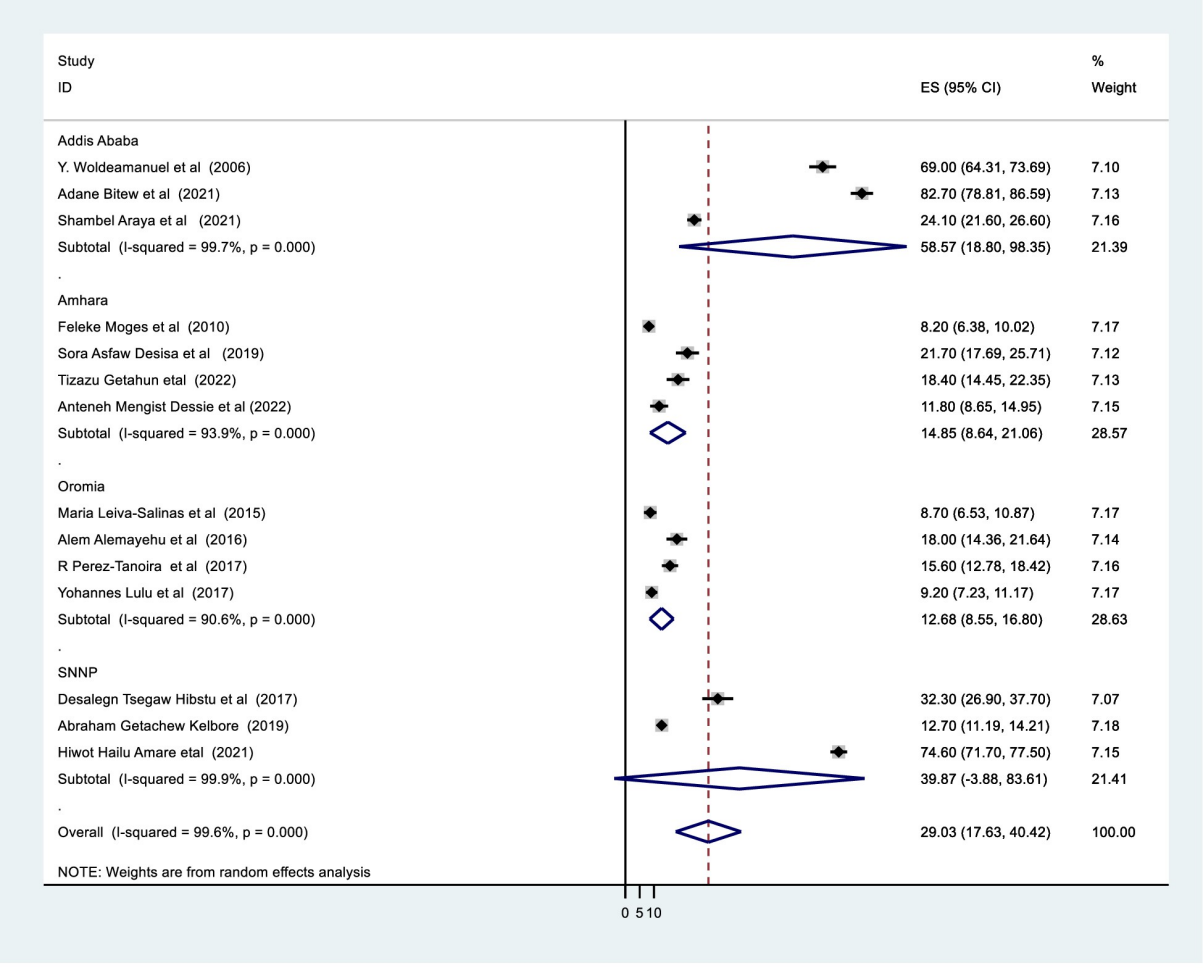
Subgroup meta analysis of tinea capitis using study setting in Ethiopia.

In Ethiopia, the prevalence of tinea capitis among primary schoolchildren was 29.03% (95%CI: 17.63–40.42). When we looked at it by region, Addis Ababa city administration had the highest prevalence of tinea capitis at 58.57 percent, followed by the southern nation and nationality of people at 39.87 percent, and Oromia regional state had the lowest prevalence at 12.68 percent (Fig 3).

### Subgroup meta–analysis

**Fig 4:**
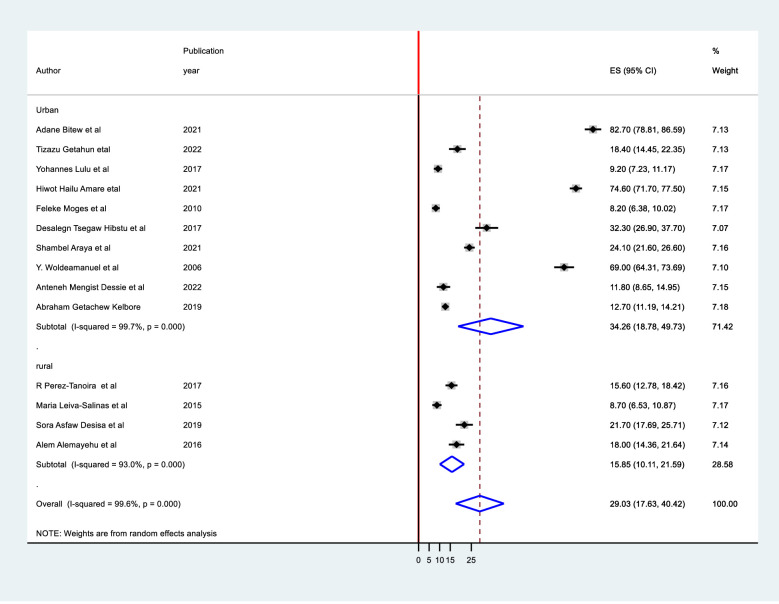
Subgroup meta analysis of tinea capitis over publication year.

**Fig 5:**
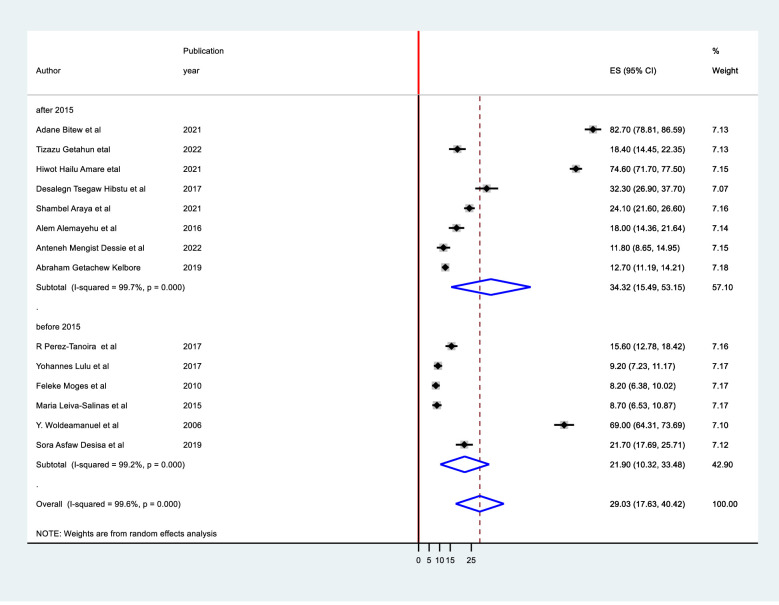
Subgroup meta nalysis of tinea capitis on sample size of Ethiopian schoolchildren.

**Fig 6:**
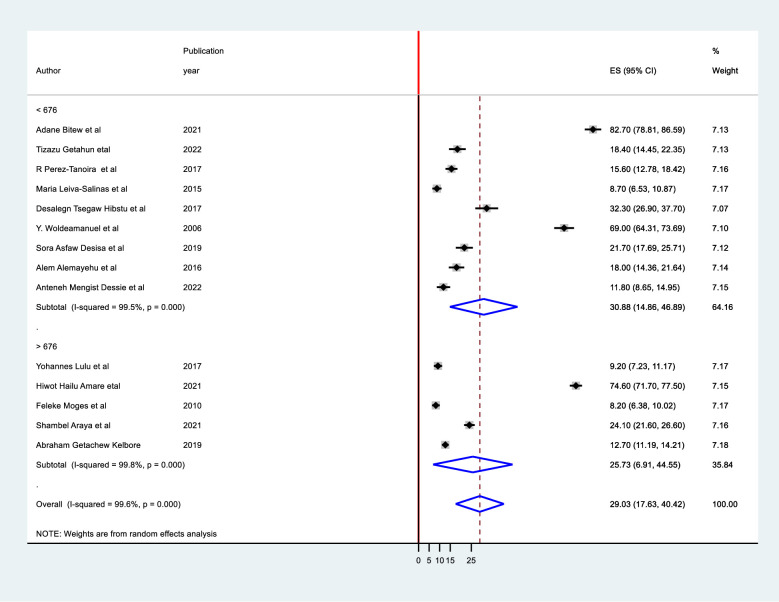
Funnel plot of tinea capitis to check publication bias.

It was computed using study setting, region, sampling method, sample size, and study period. It was discovered that there were no identified sources of heterogeneity identified using subgroup analysis despite the presence of strong evidence supporting the existence of heterogeneity (means study setting I^2^ = 99.6 with p-value 0.0001 (Fig 4), region I^2^ = 99.6 with p-value 0.001, sampling method I^2^ = 99.6, and publication year I^2^ = 99.6 with p-value 0.0001(Fig 5), sample size I^2^ = 99.8 with p-value 0.0001) (Fig 6).

### Meta-regression

The publication year and sample size were used as covariates in random-effects meta-regression. The analysis revealed that sample size (p = 0.603) and publication year (p = 0.440) had no effect on heterogeneity (Table 2).

**Table 2:** Meta-regression using publication year and sample size for tinea capitis in Ethiopian schoolchildren.

logrr	Coefficient	Std. err.	t	P>|t|	[95% conf. interval]
Year of publication	0.073	0 .09	0.80	0.44	-0.127, 0 .272
Sample size	-0.000	0.000	-0.53	0.45	-0.001, 0.001
Constant	-143.563	182.92	-0.78	0.45	-546.164, 259.034

### Publication bias (Bias detection)

**Fig 7:**
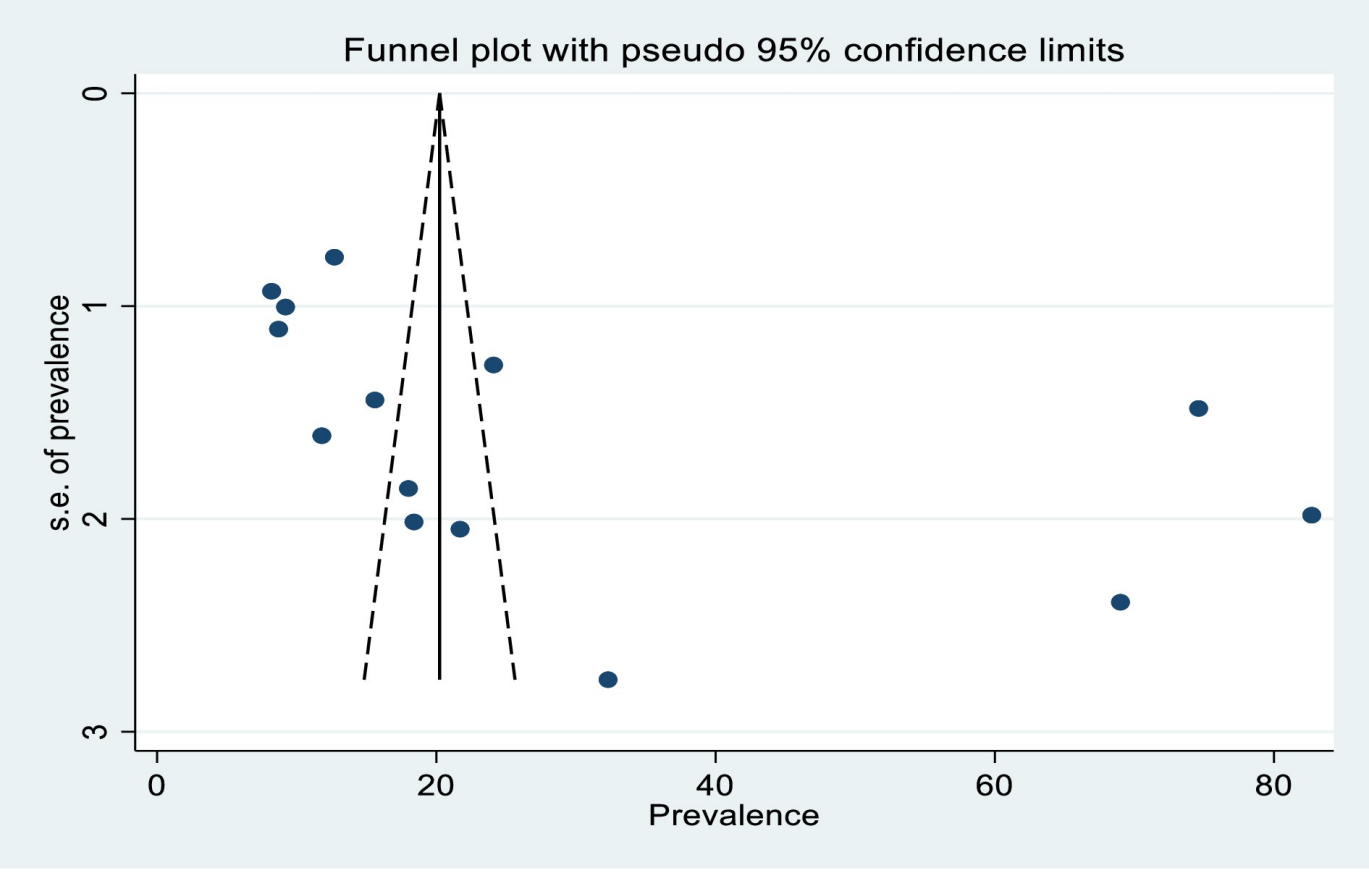
The contour _enhanced funnel plot.

**Fig 8:**
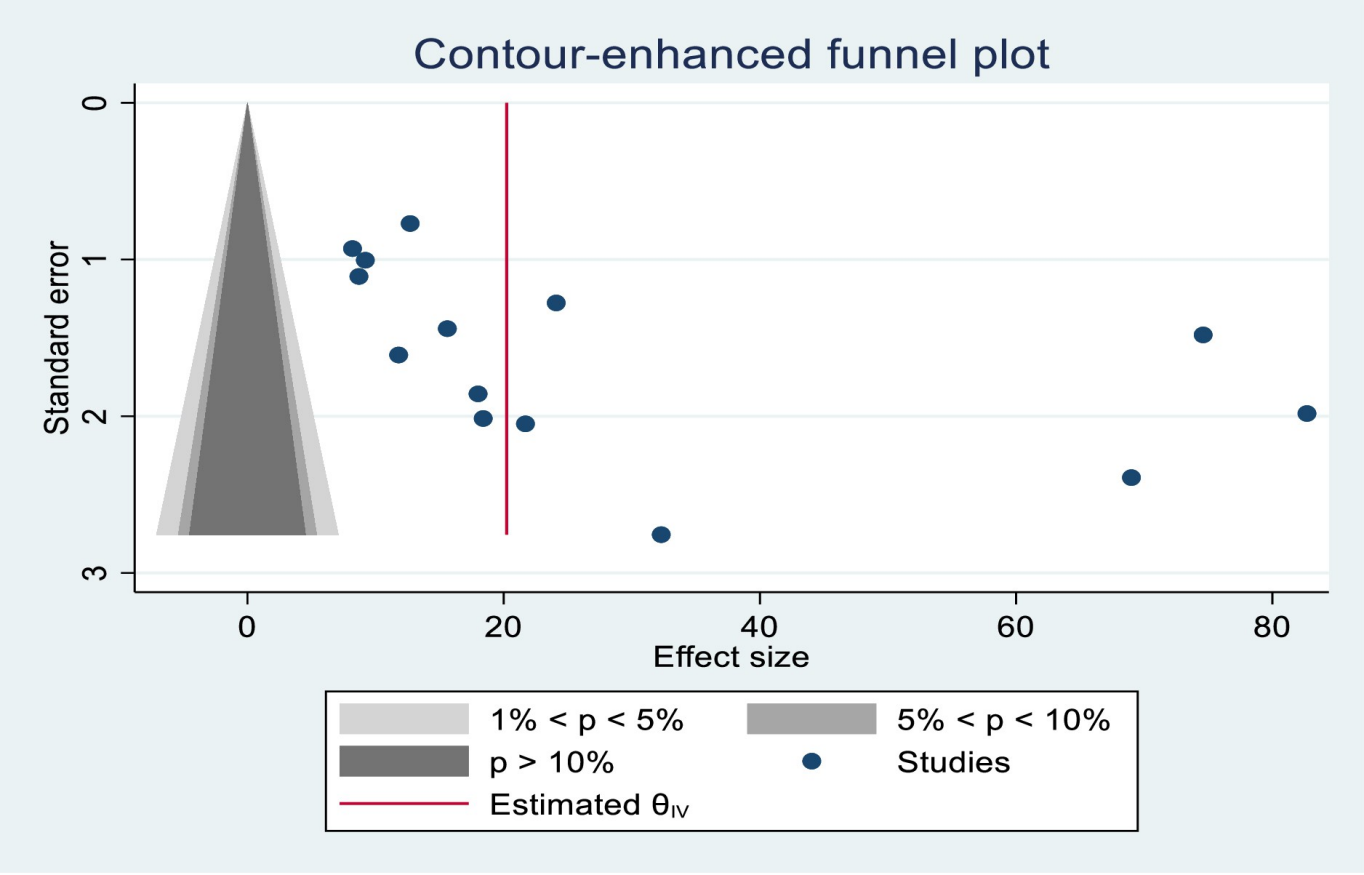
Association of tinea capitis with age of schoolchildren.

A funnel plot was used to determine the presence or absence of publication bias, and the scatter plots were asymmetrical, indicating that the small-study had effects on the heterogeneity of tinea capitis prevalence among Ethiopian primary schoolchildren (Fig 7), and contour-enhanced funnel plots were used to see if the plot asymmetry could be attributed to publication bias, and the contours show the p-value level where there is strong evidence for the presence of publication bias (Fig 8). In addition to contour-enhanced funnel plots, the egger linear regression test was used to objectively assess the presence or absence of publication bias. As a result, there was statistically significant publication bias (P = 0.040) (Table 3).

**Table 3:** The table to chek the publication bias objectively.

Std_Eff	Coefficient	Std. err.	t	P>|t|	[95% conf. interval]
Slope	-9.84	13.96	-0.71	0.49	-40.25 20.56
Bias	24.17	10.50	2.30	0.040	1.3034 47.04

### Factors associated with tinea capitis

Factors associated with tinea capitis in schoolchildren was determined based on sex (including five studies) [19,47,48,50,51], age (including four studies) [19,42,47,48], frequency of shower(including three studies) [42,46,47], frequency of hair wash(including two studies) [42,47], sharing of combs(including four studies) [20,44,46,47], and family history of tinea capitis(including three studies) [20,42,48]. As a result tinea capitis was associated with age, family history of tinea capitis, and weekly hair washing practice.

Being male had a 1.09 (95% CI: 0.56, 2.13), taking shower atleast once a week had a 0.69 (95% CI: 0.45–1.06), and sharing of comb had a 1.89 (95% CI: 0.59–6.06) times larger chance of experiencing tinea capitis than their corresponding counterparts. However, these differences were not statistically significant.

**Fig 9:**
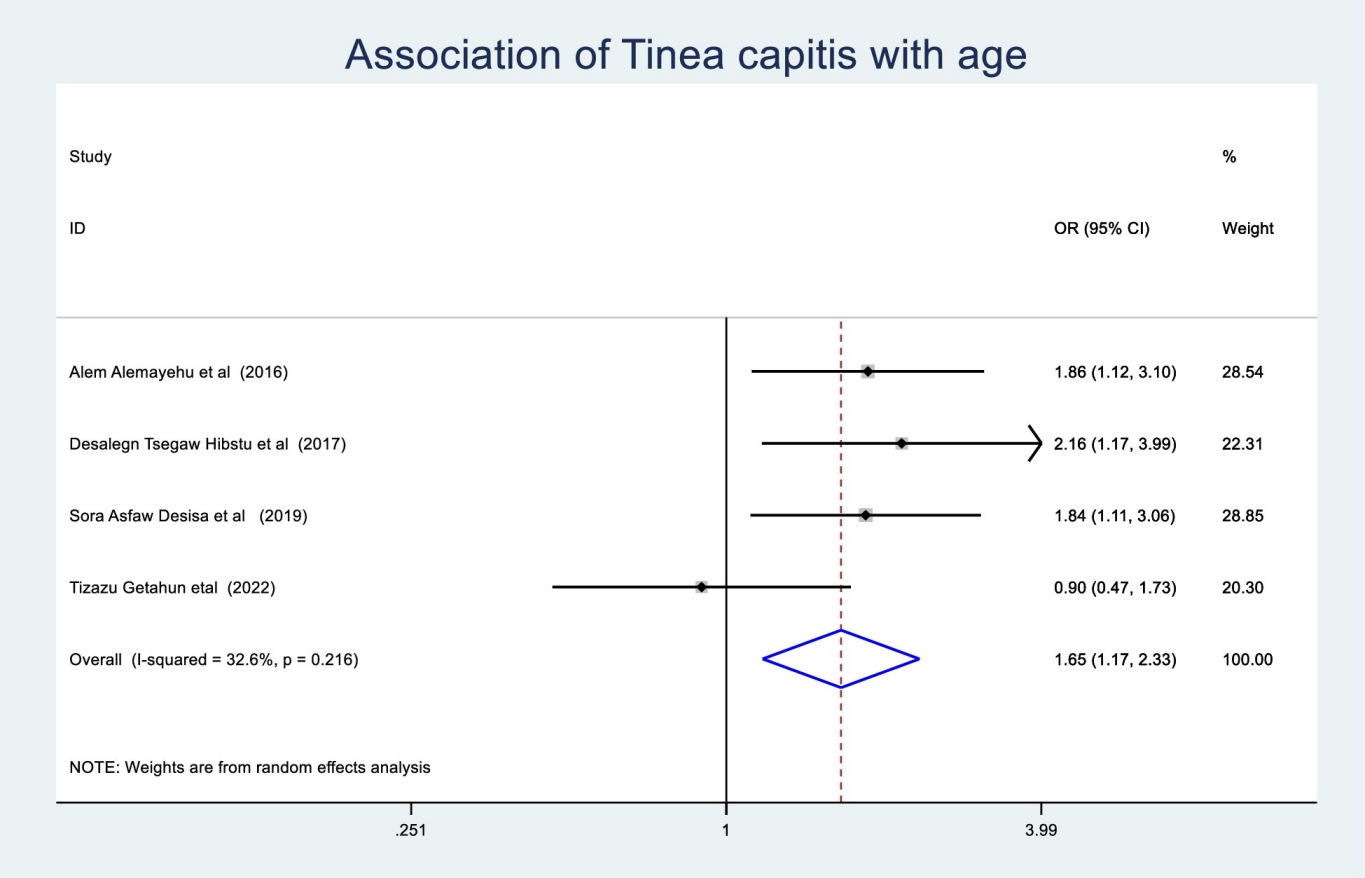
Association of tinea capitis with hair washing experience of schoolchildren.

**Fig 10:**
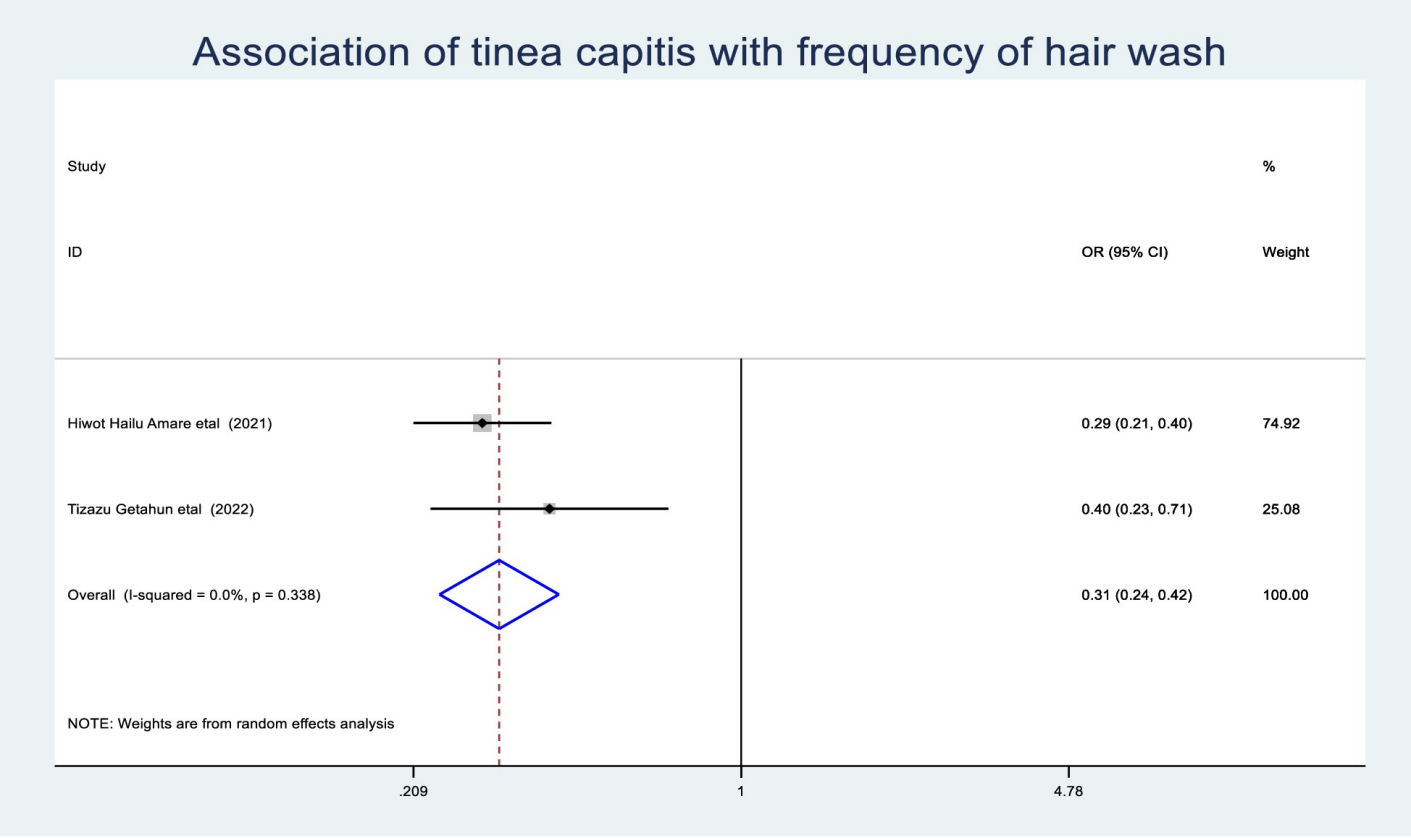
Association of tinea capitis with family history of schoolchildren.

**Fig 11:**
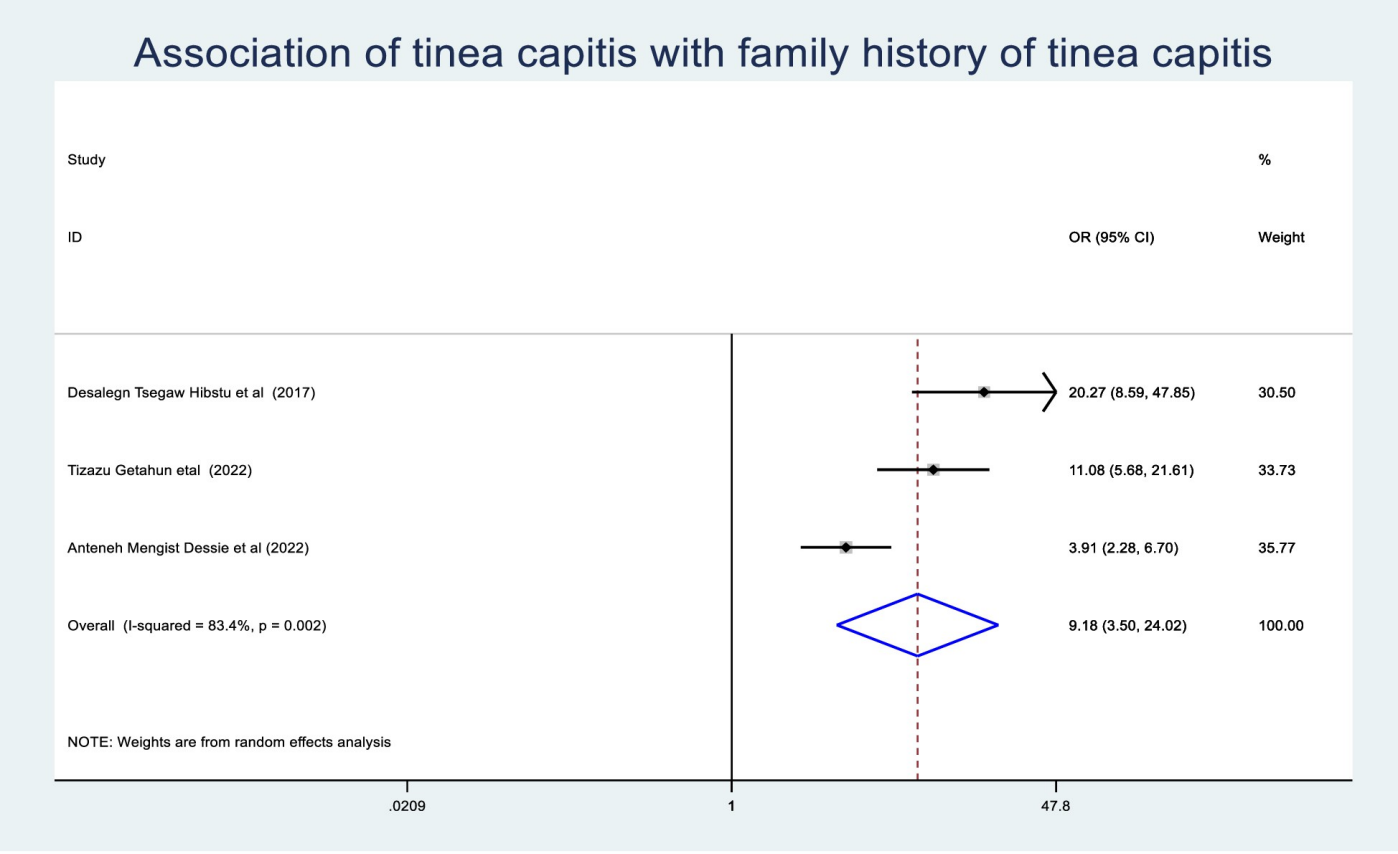
Association of tinea capitis with family history of schoolchildren.

The odds of tinea capitis among those schoolchildren who were under the age of 10 years were 1.65(95% CI: 1.17–2.33) times greater than those schoolchildren who were greater than 10 years (Fig 9). Schoolchildren who had hair wash at least once a week had a 0.31 (95% CI: 0.24–0.42) times lower chance of developing tinea capitis than schoolchildren who washed their hair more than a week (Fig 10). Schoolchildren who had family history of tinea capitis had a 9.18 (95% CI: 3.5–24.02) times greater chance of developing tinea capitis than students who had no family history of tinea capitis (Fig 11).

## Discussion

This systematic review and meta-analysis incorporated 14 articles conducted between 2006 and 2022 with 9465 schoolchildren. Each study’s sample size ranged from 288 to 1869 schoolchildren.The study participants were selected using a simple and systematic random sampling approach in each study.

The pooled prevalence of tinea capitis among schoolchildren in Ethiopia was 29.03% (95%CI: 15.37–42.71). which is consistent with a study carried out in Africa (23%) among schoolhildren conducted through a systematic review and meta analysis [30] and epidemiological trends of tinea capitis in Northen California (23.7%) [4]. Schoolchildren may feel ashamed and abashed due to the impact of tinea capitis on the external features like unsightly appearance of their scalp, leading to poor psychosocial interaction with their classmates and the society at large. Secondary bacterial infection can occur in tinea capitis patients due to the presence of moist or occluded skin, significant erosions, ulceration, or malodor, which may leads to hospitalization due to bacterial comorbidity. As a result of poor psychosocial interaction and secondary bacterial infection, students become ineffective in their education and have a low quality of life in general.

Tinea capitis affected more schoolchildren in Ethiopia than it did in lilongwe urban, Malawi among elementary schoolchildren which was 14.6% [53]. This could be as a result of the fact that maintaining personal hygiene (since it prevents the spreading of the causative agent in an individual who are already infected as well as the cross transmission of it) of schoolchildren and betterment of socioeconomic (due to a better health seeking habits) standing among lilongwe urban, Malawi parents relative to Ethiopian parents could reduce the prevalence of tinea capitis in lilongwe urban, Malawi schoolchildren compared to Ethiopian schoolchildren.

The burden of tinea capitis in Ethiopia is higher than the disease in Nigeria (14.3%) [54], Tanzania (15.3%) [55]. This may be the diagnosis approach used in Ethiopian research, which mostly rely on physical examinations alone and rarely direct microscopic examination using standard potassium hydroxide (KOH), as opposed to the culture investigation used in the Indian study, which is the gold standard examination method for fungal infections due to its high sensitivity and specificity [11]. Due to a lack of the gold standard laboratory diagnosiswhich is usedd to confirm for rule out other dermatological abnormalities, but using of physical diagnosis alone or/and direct microscopic examination having a high sensitive KOH method has/have a propensity to include any dermological disorder as tinea capitis. This could be the cause of the tinea capitis being more prevalent among Ethiopian schoolchildren as compared to Indian.

The odds of experiencing tinea capitis among schoolchildren who had family history of tinea capitis were 9.18 (95%CI: 3.5–24.02) times greather than their counterparts. This is consistent with the study conducted in Nigeria [56]. This might be, Microsporum and Trichophyton the most common etiology of tinea capitis can viable on the shared materials like towel, comb, and pillow for long periods and schoolchildrens are known by sharing their parents towel,comb, and pillow [57]. This could cause tinea capitis to spread from students’ parents to schoolchildren, and students with a family history of tinea capitis were more likely to experience the disease than their peers.

The odds of experiencing tinea capitis among schoolchildren who were under the age of 10 years old were 1.65 (95%CI: 1.17–2.33) times greater than those schoolchildren who were greater than 10 years old. This is supported by the study conducted at Southern Ethiopia [48]. This could be due to younger children having poor personal hygien practice secondary to having less knowledge and a poor attitude in order to prevent and control communicable diseases in general and tinea capitis in particular [58] as well as a lack of saturated fatty acids, which act as a natural anti-dermatophytosis mechanism [59].

The odds of experiencing tinea capitis among schoolchildren who had hair wash at least once a week were 0.31 (95%CI: 0.24–0.42) times less than those schoolchildren who had hair wash greater than a week. This consistent with the study conducted in Iraq [60]. This could be due to the fact that keeping good personal hygiene like hair washing is one method of preventing tinea capitis. So study participants who wash their hair at least once a week using soaps or shampoos which has active antifungal ingredient such as ketoconazole or selenium sulfide,can prevent tinea capitis [61]. As a result, schoolchildren who was their hair at least once a week had less tinea capitis than their counterparts. This study’s pooled prevalence of tinea capitis may not exactly represent the burden of Ethiopian schoolchildren because it was conducted using primary studies only from Amhara, South nation nationalities and people, Oromia, and Addis Ababa. In addition to the foregoing, this study only included articles published in English, which may affect the prevalence of tinea capitis and taken as a limitation of this study.

### Conclusion

One of the most prevalent childhood health condition in Ethiopia is tinea capitis, which affects over one in every four schoolchildren. Having family history of tinea capitis and under the age of 10 years old were the identified risk factors and having hair wash at least once a week was the protective factor of tinea capitis. Clinical and public engagement activities are needed to address the burden of this disease. Public engament activities like providing health education for parents and students to promot good hygiene and clinical engament activities like early diagnosis and treatment of tinea capitis need to be given great emphasis.

## Supporting information

S1 File PRISMA 2020 checklist. (DOCX)

S2 File Newcastle-Ottawa Quality assessment scale (NOQAS). (DOCX)
