# Supplementary material for: Tinea capitis among schoolchildren in Ethiopia: A systematic review and meta analysis
Source: PLoS One. 2023 Feb 10;18(2):e0280948. doi: 10.1371/journal.pone.0280948 (PMC9916598; doi:10.1371/journal.pone.0280948)
Supplement: S2 File — (DOCX) [file pone.0280948.s002.docx]

**Supplementary file 2:** The methodological quality assessment Newcastle - Ottawa Scale tool (NOS)

| **1^st^ author**  **[reference]** | **Criteria** | | | | | | | | |  |
| --- | --- | --- | --- | --- | --- | --- | --- | --- | --- | --- |
|  | **Selection** | | | | | **Comparability** | | **Outcome** | |  |
|  | Study Design | Representativeness of the sample | Sample size | Non –responders | Ascertainment of exposure/risk factor | The study controls for the most important factor | The study control for any additional factor | Assessment of the outcome | Statistical test | Quality score |
| Adane Bitew *et al* [28] | Cross-sectional | A* | A* | B* | B* | - | - | A* | **A*** | **7** |
| Tizazu Getahun *etal* [17] | Cross-sectional | A* | A* | B* | B* | - | - | A* | **A*** | **7** |
| R Perez-Tanoira *et al* [22] | Cross-sectional | B* | B* | B* | B* | - | - | A* | **A*** | **7** |
| Yohannes Lulu *et al* [23] | Cross-sectional | B* | A* | B* | A* | B* | B* | A* | **A*** | **8** |
| Hiwot Hailu Amare *etal* [25] | Cross-sectional | A* | A* | A* | B* | - | - | A* | **A*** | **7** |
| Feleke Moges *et al* [18] | Cross-sectional | A* | A* | A* | B* | B* | - | A* | **A*** | **8** |
| Maria Leiva-Salinas *et al* [31] | Cross-sectional | A* | A* | A* | B* | - | - | A* | **A*** | **7** |
| Desalegn Tsegaw Hibstu *et al* [26] | Cross-sectional | A* | A* |  | A* | B* | - | A* | A* | **8** |
| Shambel Araya *et al* [29] | Cross-sectional | A* | A* | A* | A* | B* | - | A* | A* | **8** |
| Y. Woldeamanuel *et al* [30] | Cross-sectional | A* | A* | A* | A* | B* | - | A* | A* | **8** |
| Sora Asfaw Desisa *et al* [19] | Cross-sectional | A* | A* | A* | A* | B* | - | A* | A* | **8** |
| Alem Alemayehu *et al* [24]  Anteneh Mengist Dessie *et al*[20] | Cross-sectional | A* | A* | A* | A* | B* | - | A* | A* | **8** |
| Abraham Getachew Kelbore [27] | Cross-sectional | A* | A* | A* | A* | B* | - | A* | A* | **8** |

Selection: (Maximum 5 stars)
1) Representativeness of the sample: a) Truly representative of the average in the target population. * (all subjects or random sampling) .b) Somewhat representative of the average in the target population. * (nonrandom sampling) .c) Selected group of users.d) No description of the sampling strategy.
2) Sample size:a) Justified and satisfactory. *.b) Not justified.
3) Non-respondents: a) Comparability between respondents and non-respondents characteristics is
established, and the response rate is satisfactory. * .b) The response rate is unsatisfactory, or the comparability between respondents
and non-respondents is unsatisfactory. c) No description of the response rate or the characteristics of the responders and
the non-responders.
4) Ascertainment of the exposure (risk factor): a) validated measurement tool. ** .b) Non-validated measurement tool, but the tool is available or described.* c) No description of the measurement tool.
Comparability: (Maximum 2 stars)
1) The subjects in different outcome groups are comparable, based on the study design or analysis. Confounding factors are controlled. a) The study controls for the most important factor (select one). * b) The study control for any additional factor. *
Outcome: (Maximum 3 stars)
1) Assessment of the outcome: a) Independent blind assessment. **,b) Record linkage. **,c) Self report. *,d) No description.
2) Statistical test:a) The statistical test used to analyze the data is clearly described and appropriate, and the measurement of the association is presented, including confidence intervals and the probability level (p value). *,b) The statistical test is not appropriate, not described or incomplete
